# Complication, Technical Pitfalls, and Strategies for Reducing Donor-Site Morbidity Following Connective Tissue Graft Harvesting: A Narrative Review

**DOI:** 10.3390/jcm15218739

**Published:** 2026-11-09

**Authors:** Agnieszka Droździk, Dominika Cichońska, Mariusz Lipski, Aida Kusiak

**Affiliations:** 1 Laboratory of Preclinical Periodontology, Pomeranian Medical University in Szczecin, Powstańców Wlkp. 72, 70-111 Szczecin, Poland; 2 Department of Periodontology, Medical University of Gdańsk, Marii Skłodowskiej-Curie 3C, 80-210 Gdańsk, Poland; dominika.cichonska@gumed.edu.pl (D.C.); akusiak@gumed.edu.pl (A.K.); 3 Department of Preclinical Conservative Dentistry and Preclinical Endodontics, Pomeranian Medical University in Szczecin, Powstańców Wlkp. 72, 70-111 Szczecin, Poland; mariusz.lipski@pum.edu.pl

**Keywords:** connective tissue graft, palatal donor site, postoperative complications, postoperative morbidity

## Abstract

Connective tissue grafts (CTGs) are widely used in periodontal and peri-implant plastic surgery, but palatal harvesting is associated with donor-site morbidity. This narrative review summarizes complications, technical pitfalls, and risk-reduction strategies related to subepithelial connective tissue grafts and de-epithelialized gingival graft harvesting. Subepithelial techniques enable primary closure but require adequate residual flap thickness and controlled dissection, whereas de-epithelized grafts provide better control of graft dimensions but leave an open palatal wound. Donor-site morbidity is influenced by patient-related factors, palatal anatomy, graft size, wound configuration, and surgical execution. Pain and bleeding are the most frequently reported early outcomes, while dehiscence, necrosis, infection, sensory disturbances, and symptomatic scarring remain poorly quantified. Risk may be reduced through careful assessment, minimal effective graft dimensions, atraumatic technique, reliable hemostasis, wound protection, and clear postoperative instructions. Standardized CTG-specific outcome measures and risk-assessment tools are needed to improve evidence comparability and clinical decision-making.

## 1. Introduction

Connective tissue grafts (CTGs) are among the most well-established approaches to soft-tissue augmentation in periodontal and peri-implant plastic surgery. Their clinical applications include gingival recession coverage, correction of soft-tissue defects, gingival phenotype modification, and interdental papilla reconstruction. These procedures particularly aim to improve esthetic outcomes, increase tissue thickness, and enhance the long-term stability of treatment results [1,2,3,4].

However, their principal biological disadvantage is the creation of a second surgical wound at the palatal donor site. Although alternative biomaterials may reduce or eliminate the need for autogenous tissue harvesting, their indications, predictability, and long-term performance remain highly dependent on the clinical scenario [5].

**Figure 1:**
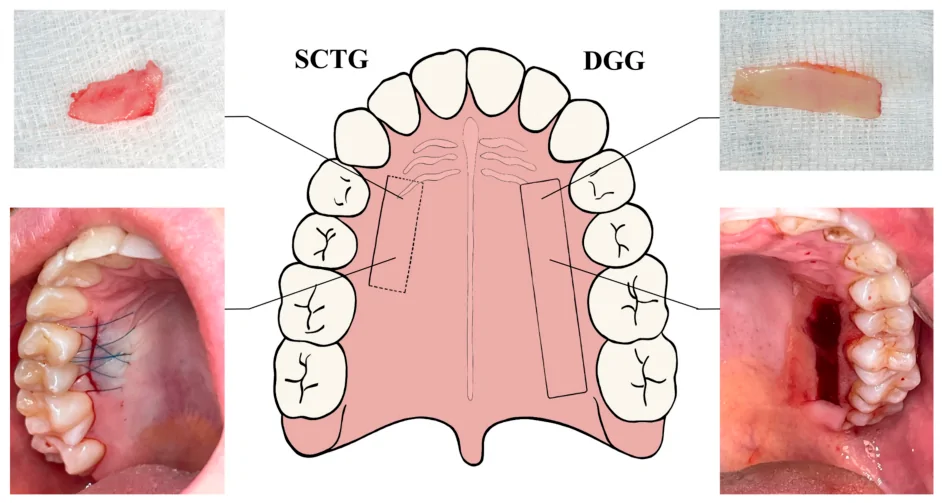
Schematic illustration of the harvesting of a subepithelial connective tissue graft and a de-epithelialized gingival graft from the palate. Clinical photographs were taken and illustrations were created by A.D.

In this review, CTGs are defined as connective tissue grafts harvested from the palate using one of two approaches: split-flap preparation to obtain a subepithelial connective tissue graft (SCTG) or de-epithelialization to obtain a de-epithelialized gingival graft (DGG) (Figure 1).

These techniques, which have the strongest evidence base, differ not only in terms of surgical access and graft preparation but also in the extent of wound exposure, control over graft thickness, and, importantly, their subsequent wound-healing patterns. Consequently, SCTG and DGG harvesting should not be regarded as equivalent methods with respect to donor-site complications and morbidity [6,7]. Harvesting techniques involving alternative donor sites, such as the maxillary tuberosity, are beyond the scope of this review. The maxillary tuberosity differs from the lateral palate in terms of tissue composition and anatomical characteristics [7].

The present review focuses on complications associated with graft harvesting from the anterolateral and posterolateral regions of the palate, which are the most frequently used donor regions. Within these regions, anatomical variability, differences in mucosal thickness and tissue composition, the location of the greater palatine foramen, and the course of the greater palatine artery define the practical limits of safe palatal tissue harvesting [8,9,10,11].

In this review, donor-site morbidity refers to the local postoperative burden attributable to the palatal donor wound, including pain, bleeding, functional discomfort, sensory disturbances, and other patient-reported impairments. Mild pain, transient discomfort, minor bleeding, and temporary impairment of eating and speaking may represent expected consequences of palatal surgery. By contrast, clinically relevant complications are unexpected events that are more appropriately defined by their intensity, persistence, progression, or the need for additional clinical intervention [7,12].

Although published systematic reviews have addressed palatal donor-site morbidity and various interventions aimed at protecting the donor site [13,14,15,16], limited guidance remains available on interpreting complications as clinical events arising from the interplay of anatomical characteristics, surgical techniques, wound exposure, patient-related factors, and postoperative care.

A narrative review is therefore justified because it allows these elements to be integrated into a clinically oriented framework rather than evaluated as isolated variables. Accordingly, this review aims to identify modifiable risk factors, clarify the mechanisms underlying donor-site complications, and outline principles for reducing donor-site morbidity and preventing and managing complications following palatal CTG harvesting.

## 2. Objective and Review Approach

The objective of this narrative review is to critically synthesize the current evidence and clinically relevant concepts concerning complications, technical errors, and strategies for reducing donor-site morbidity following palatal connective tissue graft harvesting. The review focuses on donor-site complications associated with SCTG and DGG harvesting. Studies on free gingival grafts were included only when their findings were relevant to palatal donor-site morbidity, wound healing, hemostasis, or donor-site protection.

A literature search of full-text English-language publications was conducted in three electronic databases—PubMed, Scopus, and Web of Science—and supplemented by searches in Google Scholar. The search covered the period from 1974, when subepithelial connective tissue graft harvesting was first described in the literature, to the end of May 2026. The reference lists of key publications were also screened manually.

Search terms included combinations of the following terms: “connective tissue graft”, “subepithelial connective tissue graft”, “de-epithelialized graft”, “palatal donor site”, “donor-site morbidity”, “palatal wound healing”, “postoperative pain”, “bleeding”, hemostasis”, and “palatal stent”. Publications were selected in two stages: title and abstract screening followed by full-text assessment performed independently by two authors (A.D. and D.C.).

The evidence base included umbrella reviews, systematic reviews, and narrative reviews; randomized clinical trials; prospective clinical studies; and anatomical, histological, and imaging-based investigations. The key studies included in this narrative review are presented in Table 1.

Clinical interpretation was based primarily on human studies. Cadaveric studies were included when they provided essential information on palatal anatomical relationships. The literature was organized according to anatomical factors, harvesting techniques, type of complications, preventive interventions, and management strategies.

Given the narrative nature of this review, no formal risk-of-bias assessment or meta-analysis was performed. Systematic reviews and randomized clinical trials were prioritized when evaluating palatal neurovascular anatomy, patient-reported outcomes, palatal wound healing and preventive interventions. Observational studies were considered particularly relevant to surgical safety and the assessing the composition and thickness of palatal tissues.

Because donor-site morbidity is often evaluated using heterogeneous patient-reported and wound-healing outcomes, numerical results were interpreted within their specific clinical context rather than directly compared across studies. When the available evidence was limited or conflicting, conclusions were presented as clinical interpretations rather than definitive recommendations. Accordingly, this article should be regarded as a narrative synthesis that integrates the available evidence on palatal connective tissue graft harvesting and considers its implications for everyday clinical practice.

## 3. Palatal Anatomy and Histological Characteristics Relevant to CTG Harvesting

The anatomical characteristics of the hard palate are critical to understanding the technical errors and complications associated with connective tissue graft harvesting. Current evidence suggests that donor-site morbidity is determined not only by the type of harvesting technique and its execution, but also by the complex interplay among palatal mucosal thickness, graft dimensions, and the course of the greater palatine neurovascular bundle (GPB) [8,21]. Both graft quality and donor-site morbidity are substantially influenced by the layered architecture of the palatal tissues and regional variations in palatal mucosal thickness [8]. The palate should not be considered a homogeneous donor site.

### 3.1. Histological Architecture of the Palatal Mucosa

Histologically, the palatal masticatory mucosa consists of keratinized stratified squamous epithelium, lamina propria, and submucosa. The orthokeratinized epithelium, which is 0.24–0.41 mm thick, forms the outer protective layer. The lamina propria beneath it comprises two distinct layers: a papillary layer with a greater cell density and a reticular layer containing thicker collagen bundles. These differences may be clinically relevant and graft orientation during the procedure may be important. The thickness of the lamina propria is relatively consistent throughout the palate, except in the region of the maxillary tuberosity, where it is greater. The underlying submucosa contains blood vessels, nerves, adipose tissue, and glandular elements. When the epithelium is excluded, the lamina propria and submucosa account for approximately 37% and 63% of the tissue volume, respectively [29,30]. It has been suggested that graft composition, particularly the relative proportions of dense fibrous connective tissue, adipose tissue, and glandular components, may influence graft integration, revascularization, shrinkage and the subsequent tissue response at the recipient site [8,23].

From a graft-harvesting perspective, the lamina propria is the most relevant component. Excessively superficial harvesting may produce a thin or epithelium-rich graft, whereas excessively deep harvesting increases donor-site trauma and may incorporate a greater proportion of submucosal tissue. The relative proportions of fibrous connective tissue and adipose or glandular components within CTG specimens depend on both the donor region and the harvesting technique [8,31,32].

### 3.2. Palatal Mucosal Thickness and Residual Tissue Dimensions

Palatal mucosal thickness exhibits substantial interindividual and site-specific variability. Reported mean values range from approximately 1.8 to 3.9 mm, depending on the anatomical region and measurement method, as discussed in Section 5 (Preoperative Issues). In general, tissue thickness tends to increase from the gingival margin toward the palatal midline and from the canine toward the molar region, although localized thinning may occur near the palatal root prominence of the first molar [33].

Importantly, greater tissue thickness does not necessarily indicate a more favorable donor site, because grafts harvested from thicker palatal mucosa may contain a larger proportion of adipose or glandular tissue rather than the desired dense lamina propria [8]. Palatal tissue thickness influences postoperative morbidity by determining the amount of tissue remaining after graft harvesting. In DGG harvesting, this refers to the thickness of the tissue remaining over the underlying bone, whereas with SCTG harvesting, the thickness of the residual palatal flap appears to be more relevant. The residual flap should ideally be at least 1 mm thick to minimize the risk of flap sloughing and necrosis, both of which may increase postoperative discomfort [6,22].

### 3.3. Greater Palatine Neurovascular Anatomy

The greater palatine neurovascular bundle represents the principal anatomical limitation during palatal graft harvesting. The greater palatine artery (GPA) and nerve emerge through the greater palatine foramen, which is most commonly located at the midpalatal aspect of the maxillary third molar (57–70% of cases), approximately 8 mm from the margin of the alveolar process. After emerging from the foramen, the greater palatine artery courses anteriorly toward the incisive foramen. Beyond the bony prominence, it typically gives rise to medial and canine branches. The greater palatine nerve runs medial to the artery. The distance between the GPA and the cementoenamel junction (CEJ) varies along the palate. This distance is generally greater in the posterior region, particularly around the second molar (13.9 ± 1.0 mm), and decreases towards the canine region (9.9 ± 2.9 mm), where the vertical safety margin is reduced [9,17,34].

However, considerable anatomical variability in the branching pattern of the GPA must be considered. A clinically challenging configuration occurs when a vessel courses close to the CEJ of the posterior teeth. In this configuration, the medial branch arises from the lateral branch before the latter reaches the bony prominence, whereas the canine branch emerges immediately beyond this prominence. This anatomical configuration has been reported in approximately 25% of cases [35]. Palatal vault morphology further influences this anatomical relationship, because a shallow vault may position the neurovascular bundle closer to the alveolar crest [36].

### 3.4. Clinical Definition of the Palatal Safe Zone

Taken together, the anatomical evidence supports a comprehensive and individualized assessment of the safe zone for palatal graft harvesting. This zone should be determined not only by the course of the GPA, its distance from the CEJ, and anatomical variations, but also by palatal soft-tissue thickness and donor-site morphology. Consequently, a universally defined harvesting area may not adequately account for individual anatomical variability [9,10,11].

## 4. Techniques for Connective Tissue Graft Harvesting

### 4.1. Classification and Clinical Implications

Palatal connective tissue graft harvesting techniques differ primarily in incision design, the extent of flap elevation, surgical visibility, control of graft thickness, and the possibility of primary wound closure. From a donor-site perspective, these techniques can broadly be classified into subepithelial and open-wound approaches. Subepithelial techniques aim to preserve a covering palatal flap, facilitating primary wound closure and healing by primary intention. In contrast, open-wound techniques leave the palatal donor site exposed to heal by secondary intention, which may prolong wound healing and increase postoperative discomfort [27]. This distinction is clinically relevant because it highlights the balance between surgical access, preservation of donor-site tissues, and control over graft composition when seeking to minimize donor-site morbidity and complications.

### 4.2. Histological Characteristics of SCTGs and DGGs

Histological studies indicate that subepithelial connective tissue grafts (SCTG) are less dense and contain a smaller proportion of lamina propria than de-epithelialized gingival grafts (DGGs). They have a greater adipose and glandular tissue content, may make SCTGs less dimensionally stable and may act as a barrier to vascularization during the initial phase of healing. By contrast DGGs appear firmer, less susceptible to postoperative shrinkage, and easier to handle [6,8]. However, their denser structure may make them more susceptible to necrosis than SCTGs [21]. Additionally, epithelial remnants have been reported [32]. The amount of tissue available and tissue structure characteristic of grafts depend on the donor site. Consequently, the selection of the donor region should be guided by the intended clinical application [21].

Several techniques have been proposed for harvesting CTGs from the lateral palate. Because the available evidence does not establish the superiority of any single approach, the following section provides a brief historical overview of these techniques, highlighting how they evolved, why their wound configurations differ, and how these differences may influence donor-site morbidity.

### 4.3. Subepithelial Connective Tissue Graft Techniques

The development of subepithelial connective tissue graft (SCTG) harvesting techniques began with the trap-door approach (TD) described by Edel in 1974 [37]. In 1982, Langer and Langer introduced the use of CTGs for root coverage [38]. Subsequent modifications aimed to reduce the number of incisions and surgical trauma, including the double-incision technique described by Bruno in 1994 [39] and the single-incision technique introduced by Hürzeler and Weng in 1999 [40]. These approaches differ in surgical access, graft preparation, and the potential for primary wound closure. Selected milestones and their implications for donor-site morbidity are summarized in Table 2.

### 4.4. De-Epithelialized Gingival Graft Technique

The de-epithelialized gingival graft (DGG) represents an alternative harvesting approach, mentioned by Zucchelli et al. in 2003 and subsequently evaluated clinically in 2010 [6,44] (Table 2). It involves harvesting an epithelialized gingival graft followed by extraoral de-epithelialization, although intraoral de-epithelialization has also been described [23,47]. Unlike subepithelial techniques, DGG harvesting generally leaves an exposed palatal wound that heals by secondary intention.

Some studies have reported more favorable root-coverage outcomes with DGG combined with a coronally advanced flap [48] or tunnel technique [23]. However, a more recent systematic review and meta-analysis found no statistically significant differences between DGG and SCTG at 12 months in gingival recession reduction, complete root coverage, or gains in keratinized tissue width, gingival thickness, and clinical attachment level [49]. Given the very low certainty of the available evidence, the clinical superiority of either harvesting approach remains unestablished.

### 4.5. Selection of the Harvesting Technique

In light of the considerations outlined above, the selection of a harvesting technique requires balancing surgical access and control over graft dimensions and tissue composition against the need to minimize donor-site trauma and morbidity. Given the limited comparative evidence, the choice of harvesting technique remains substantially influenced by clinical judgment and expert opinion. Subepithelial approaches may be favored in esthetically demanding areas or when primary closure of the palatal donor site is a priority, provided that palatal mucosal thickness is sufficient to preserve a viable covering flap. In contrast, a de-epithelialized gingival graft may be feasible even in relatively thin palatal mucosa. This approach may also offer advantages when larger graft dimensions or greater control over graft thickness and tissue composition is desired [1,23].

## 5. Preoperative Issues

Preoperative errors in palatal connective tissue graft harvesting may occur when clinically relevant risk factors are not identified or adequately incorporated into the surgical plan. These potential errors can be grouped into four principal domains: medical risk assessment; anatomical evaluation and technique selection; graft dimensions planning; and informed consent, patient preparation, and morbidity-reduction strategies.

### 5.1. Preoperative Risk Assessment of Systemic Conditions and Smoking

The most clinically relevant preoperative oversights are those that may compromise hemostasis or the biological conditions necessary for palatal wound healing. Anticoagulant and antiplatelet therapy should not be regarded as automatic contraindications to palatal connective tissue graft harvesting. The key issue is the individualized assessment of thromboembolic and local bleeding risk, considering the type of medication, indication for therapy, stability of the underlying condition, history of bleeding history, anticipated wound extent, and feasibility of achieving local hemostasis. Therefore, the error lies not necessarily in performing surgery in a patient receiving these medications, but in failing to establish and document an appropriate hemostatic strategy or, when indicated, to consult the treating physician [50].

The need for individualized assessment also applies to systemic conditions that may impair wound healing through their effects on perfusion, oxygen delivery, inflammatory regulation, fibroblast activity, collagen metabolism, or resistance to infection. These include diabetes mellitus, immunosuppression, hematologic disease, liver disease, vascular disorders, uncontrolled hypertension, and disorders affecting collagen turnover. However, risk thresholds specifically associated with complications following connective tissue graft harvesting remain poorly defined [25,50,51].

Smoking should also be documented as a modifiable risk factor because it may adversely effect vascularity, tissue oxygenation, fibroblast function, and collagen synthesis. Although direct evidence on smoking and palatal donor-site healing after CTG harvesting remains limited, smokers have been reported to exhibit an approximately 25–30% lower blood vessel density in subepithelial connective tissue grafts than nonsmokers. This finding supports the biological plausibility that reduced vascularity may impair tissue nutrition, delay revascularization, and negatively influence wound repair [51].

### 5.2. Preoperative Assessment of Palatal Anatomy

Another potential preoperative error is inadequate assessment of palatal anatomy. The palate should not be considered a uniform donor site because mucosal thickness, tissue composition, and the course of the greater palatine neurovascular bundle vary among patients and across different regions of the same palate [9]. Preoperative planning should therefore include identification of the intended donor region, assessment of mucosal thickness, and selection of an appropriate harvesting technique based on individual anatomical characteristics and limitations.

**Figure 2:**
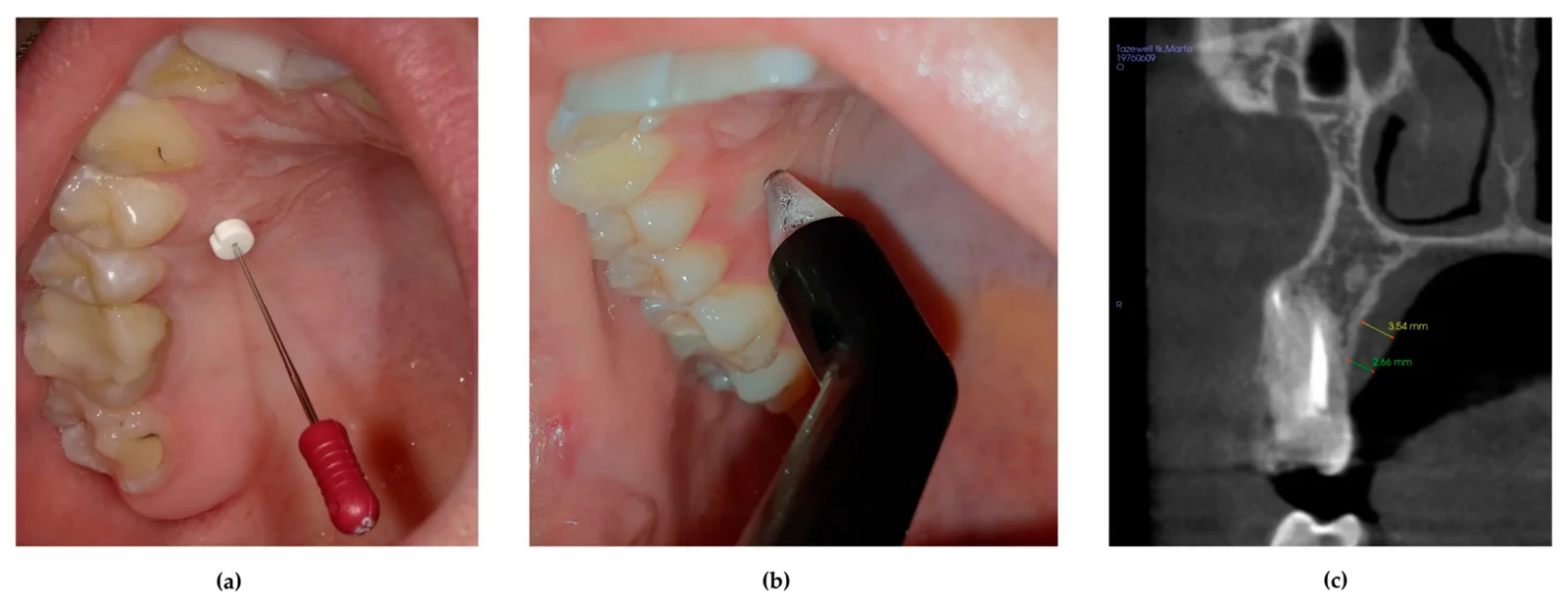
Preoperative assessment of palatal mucosal thickness using (**a**) an endodontic instrument with a silicone stopper inserted perpendicular to the mucosal surface; (**b**) the small probe head of an ultrasonic device; and (**c**) CBCT cross-sectional measurement supporting the preoperative evaluation of the available donor tissue and residual safety margin. In the presented image, obtained at the level of the maxillary second premolar, the mucosal thickness ranged from approximately 2.60 to 3.64 mm. Clinical photographs taken by A.D.

Transmucosal bone sounding using a periodontal probe or an endodontic instrument fitted with a silicone stopper (Figure 2a), performed with minimal local anesthesia, remains a commonly used chairside assessment method. Ultrasonography (Figure 2b) and cone-beam computed tomography (CBCT: Figure 2c) may be considered in selected cases, particularly when tissue thickness is uncertain, palatal vault morphology is unfavorable, or extensive graft harvesting is planned [10,11]. In anatomically challenging cases, ultrasound-guided graft harvesting has also been proposed as a promising approach [52].

### 5.3. Planning of Graft Dimensions

Inadequate planning of graft dimensions represents another avoidable preoperative error. The objective is not to harvest the largest possible graft but rather the smallest graft that can predictably meet the requirements of the recipient site. Surgical templates or preoperative measurement may facilitate accurate transfer of the required graft dimensions from the recipient site to the palate. Graft harvesting may be considered excessive when the graft dimensions exceed clinical requirements, resulting in unnecessary enlargement of the donor-site wound, or extension beyond the anatomically defined safe zone [1,21].

### 5.4. Informed Consent and Morbidity-Oriented Patient Preparation

Informed consent should be distinguished from patient preparation aimed at reducing postoperative morbidity. The informed consent process ensures that patients are informed about the procedure and its associated risks before providing their consent, whereas patient education prepares them for the expected postoperative course, appropriate self-care, and early recognition of potential complications, including abnormal bleeding, delayed healing, necrosis, and sensory disturbances. Written and verbal postoperative instructions should therefore be regarded as important elements of complication prevention, early detection, and timely management, rather than merely administrative requirements [25,53].

## 6. Intraoperative Technical Errors

Intraoperative errors during palatal connective tissue graft harvesting should be distinguished from complications and adverse events that may occur despite technically correct surgery. In this review, a technical error is defined as a modifiable deviation from appropriate surgical practice involving incision design, dissection depth, tissue handling, hemostasis, or wound stabilization. By contrast, adverse clinical outcomes, such as bleeding, delayed epithelialization, pain, necrosis, and sensory disturbances, discussed in the following sections, are clinical outcomes that may result from technical errors but may also arise from anatomical variability or patient-related risk factors [7,39]. This distinction is important because most available studies report donor-site morbidity rather than directly evaluating the associations between specific intraoperative errors and postoperative clinical outcomes.

### 6.1. Inappropriate Incision Placement

One of the principal intraoperative errors is inappropriate incision placement. The incision site should be determined based on the selected harvesting technique, the required graft dimensions, and individual palatal anatomy, including mucosal thickness and the course of the greater palatine artery. A coronal incision placed too close to the gingival margin may compromise the integrity of the remaining coronal tissue bridge and its vascular supply. Particular caution is therefore warranted in patients with periodontal disease.

Conversely, an excessively apical incision may increase the risk of injury to the GPA or its branches, especially in patients with shallow palatal vaults, in posterior donor regions, or when grafts with larger apicocoronal dimensions are required [9,10,11]. Incision placement should therefore not be based on a fixed harvesting map but should be individualized according to the patient’s anatomical characteristics and the specific surgical requirements.

### 6.2. Inadequate Control of Dissection Depth

A second clinically relevant, technique-dependent error is inadequate control over dissection depth, which may lead to harvesting at an excessively deep or superficial plane. From the recipient-site perspective, sufficient graft volume and an appropriate connective tissue composition are important for successful treatment outcomes. However, unnecessarily deep dissection into adipose, glandular, or highly vascular submucosal tissues may compromise graft quality, increase intraoperative bleeding, and expose deeper anatomical structures, thereby potentially impairing palatal donor-site healing [31,32,54].

According to Wei et al. [54], preserving a residual tissue thickness of at least 2 mm may be important for favorable long-term wound healing. In subepithelial harvesting techniques, inadequate control of the dissection depth—particularly during the operator’s learning curve—may result in excessive thinning of the overlying palatal flap. This may compromise its vascular supply and viability, potentially impairing primary wound healing and increasing the risk of flap sloughing and necrosis [6,22].

### 6.3. Intraoperative Failure to Adhere to the Planned Graft Dimensions

Deviations from the planned graft dimensions may result from inaccurate transfer of preoperative measurements to the palatal donor site. The objective is to harvest a graft that meets the requirements of the recipient site, based on direct measurements or a surgical template. Harvesting a graft that is larger or thicker than clinically necessary may increase the extent or depth of the donor-site wound, cause unnecessary tissue trauma, and require extension of the harvesting area into anatomically less favorable regions [1,21].

### 6.4. Excessive Tissue Trauma and Prolonged Operative Time

Excessive tissue trauma represents another potentially modifiable intraoperative risk factor. Repeated instrumentation, crushing of the wound margins, excessive traction, and prolonged tissue manipulation may compromise tissue viability, intensify the inflammatory response, exacerbate the inflammatory response, and ultimately delay wound healing. Optical magnification, including surgical loupes and operating microscopes, may improve visualization and precision in periodontal plastic surgery, thereby facilitating atraumatic tissue handling and precise approximation of wound margins [55].

Although several studies have reported associations between operative time and postoperative morbidity, direct evidence identifying surgical duration as an independent risk factor for palatal donor-site morbidity remains limited [25,28,56]. Nevertheless, a large cohort study assessing postoperative morbidity following various surgical procedures identified operative time, including the time required for graft harvesting, as the strongest predictor of postoperative morbidity. Each additional minute of surgery was associated with 4% higher odds of moderate-to-severe pain and 3% higher odds of moderate-to-severe swelling [25].

These findings should be interpreted cautiously, as prolonged procedures may reflect challenging anatomy, larger graft dimensions or intraoperative bleeding rather than a direct causal effect of surgical duration itself [12,57].

Operator experience and the associated learning curve may also influence surgical efficiency and operative time. Greater operator experience may contribute to improved technical performance and shorter operative times in periodontal surgery, potentially influencing postoperative morbidity [58,59,60,61]. However, to our knowledge, no studies have directly evaluated the relationship between the operator learning curve and donor-site morbidity following palatal connective tissue graft harvesting.

### 6.5. Inadequate Hemostasis and Wound Stabilization

Hemostasis-related errors include failure to identify the bleeding source, insufficient local compression, and premature wound closure despite ongoing bleeding. Persistent pulsatile bleeding, rapid reaccumulation of blood after compression, or failure to achieve stable hemostasis should raise suspicion of clinically significant vascular injury and prompt further assessment and appropriate hemostatic intervention before wound closure. The procedure should not be completed until active bleeding has been controlled and stable hemostasis has been achieved [28,57].

## 7. Intraoperative Complications

Intraoperative complications associated with palatal connective tissue graft harvesting refer to adverse events occuring during the surgical procedure. Although such events may be clinically significant, their incidence remains poorly quantified. Much of the available literature focuses primarily on postoperative pain, wound healing, wound-dressing interventions, or patient-reported morbidity rather than the systematic assessment of intraoperative complications [14,16,18]. Similarly, studies comparing harvesting techniques or hemostatic approaches provide limited standardized data on the incidence of severe intraoperative bleeding, flap perforation, and neurovascular injury [20,22,62].

### 7.1. Intraoperative Bleeding

Bleeding is one of the most clinically significant and potentially hazardous intraoperative complications associated with palatal graft harvesting. It should be assessed according to its source and severity.

**Figure 3:**
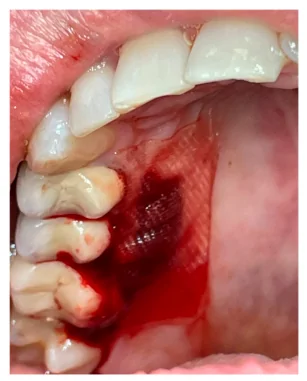
Immediate recurrent bleeding as an intraoperative complication. After initial hemostasis was achieved by local pressure and placement of an oxidized cellulose dressing, rebleeding occurred shortly thereafter. Insufficient clot stability indicates the need for reassessment and additional local hemostatic measures. Clinical photography taken by A.D.

Diffuse oozing from small vessels within the palatal mucosa is common during dissection and can generally be controlled with local hemostatic measures. These measures include direct compression with moistened gauze for approximately 5 min, followed by suturing when indicated [57,63]. Bleeding that persists despite these measures or recurs shortly after initial hemostasis (Figure 3) should be regarded as clinically significant intraoperative bleeding requiring additional hemostatic management [18].

A potentially more serious complication is direct injury to the main trunk of the greater palatine artery (GPA) or one of its branches [18,28,57]. As previously noted, the actual incidence of such injuries remains unknown. Excessive or prolonged intraoperative bleeding resulting from palatal vascular injury may substantially impair visualization of the surgical field, interrupt the procedure, and prolong operative time. Bleeding from the GPA may be difficult to control and may result in significant blood loss. Associated vascular injury may also compromise local tissue perfusion, potentially contributing to palatal tissue necrosis. Intraoperative bleeding may also increase patient anxiety and adversely affect their perception of the surgical procedure [57].

### 7.2. Flap Thinning and Perforation

Flap perforation and excessive flap thinning represent additional intraoperative adverse events associated with closed subepithelial harvesting techniques. Although these complications may occur despite careful surgical execution, they may also result from technical errors, including inappropriate dissection depth, uncontrolled dissection, or excessive traction [7,46].

From a clinical perspective, excessive flap thinning and perforation should be viewed as interrelated events. As excessively thinned flap may perforate during dissection, while flap perforation may convert a donor-site wound intended for primary closure into a partially exposed wound. This may increase bleeding and contribute to palatal tissue sloughing, although the magnitude of these effects has not been precisely quantified [7,43].

The risk of excessive flap thinning and perforation may increase when the planned graft dimensions exceed the available volume of palatal tissue, especially in patients with unfavorable palatal anatomy or insufficient mucosal thickness [12,21].

## 8. Early Postoperative Morbidity and Complications

Early postoperative complications are defined in this review as adverse events occurring within the first 7–14 days following palatal connective tissue graft harvesting. This interval encompasses clot stabilization, early epithelial repair, and a period of increased mechanical vulnerability of the palatal wound. It also corresponds to the early follow-up intervals commonly used in clinical studies [20,24,25,64]. Pain, bleeding, swelling, and transient functional limitations occurring during this period are considered indicators of early donor-site morbidity, although they do not necessarily represent complications [18]. Postoperative morbidity following connective tissue graft harvesting is relatively common but is generally mild and manageable [13]. Persistent sensory disturbances, scarring and sequelae of delayed wound healing are discussed separately in Section 10 as late donor-site complications.

Importantly, the assessment of early postoperative complications should include both clinician-assessed outcomes and patient-reported outcomes (PROs). Combining PROs with objective clinical assessments provides a more comprehensive evaluation of postoperative morbidity. This approach is particularly relevant to palatal graft harvesting because the clinical assessed severity of an adverse event may not directly correspond directly to the burden experienced by the patient. PROs therefore represent clinically meaningful endpoints in both clinical practice and research [65].

### 8.1. Postoperative Pain

Postoperative pain is the most frequently assessed early postoperative outcome and a major determinant of patient-perceived morbidity following palatal graft harvesting. It is most commonly evaluated using a visual analog scale (VAS), with analgesic consumption serving as an additional indirect measure. Pain is an expected consequence of surgery; it typically peaks on postoperative days 1–3 and subsequently subsides, usually resolving within approximately 2 weeks [13,14,18,22].

Harvesting techniques that preserve epithelial coverage and permit primary wound closure have been associated with reduced postoperative pain and discomfort [19,20,24,26,27,64]. However, when flap necrosis occurs, pain may intensify, and its severity may correlate with the extent of necrosis [6,66].

Postoperative pain and discomfort following de-epithelialized gingival graft (DGG) harvesting may be reduced by limiting graft dimensions and thickness and providing appropriate wound protection. Smaller graft dimensions—particularly an apicocoronal height of up to 4 mm and a thickness of less than 2 mm—along with preservation of a residual palatal tissue thickness of greater than 2 mm, have been associated with reduced postoperative morbidity [6,21,54,67]. Evidence regarding the influence of graft length on postoperative morbidity remains inconclusive. Although a trend toward increased discomfort with greater graft length has been reported, statistically significant associations have not been consistently demonstrated. Importantly, these findings are derived from studies involving free epithelialized grafts rather than DGG harvesting [68,69].

Adequate protection of the exposed palatal wound is another important consideration. Zucchelli et al. [6] reported no significant difference in postoperative pain between the conventional trap-door technique and DGG harvesting when the exposed donor-site wound was protected with an absorbable hemostatic dressing.

The effectiveness of adjunctive strategies aim at reducing postoperative pain and discomfort and promoting wound healing has been extensively evaluated in the literature [14,15,16]. These approaches are discussed in Section 11, Risk-Reduction Strategies for Donor-Site Morbidity and Complications.

Current evidence does not support a universal ranking of graft-harvesting techniques based on postoperative pain, despite the potential relevance of this outcome to clinical decision-making. The interpretation of the available evidence is further limited by the lack of clear differentiation between donor- and recipient-site outcomes in several studies, making it difficult to attribute reported pain and discomfort specifically to the palatal donor site [19,23,64]. An additional limitation is the heterogeneity across studies with respect to graft dimensions, residual tissue thickness, flap thickness, suturing techniques, donor-site wound protection, postoperative analgesic protocols and other aspects of postoperative care.

### 8.2. Delayed Postoperative Bleeding

Delayed postoperative bleeding, defined as bleeding that develops or recurs after completion of the surgical procedure, should be assessed according to its clinical severity. Minor oozing that resolves spontaneously or can be controlled with local pressure is relatively common and should be distinguished from clinically significant hemorrhage requiring professional intervention [19,50].

The risk and clinical presentation of palatal donor-site bleeding vary depending on the harvesting technique and the mode of wound healing. Donor sites left to heal by secondary intention after DGG or FGG harvesting may be more susceptible to postoperative bleeding [25,70]. The absence of epithelial coverage may increase the risk of mechanical clot disruption during eating, speaking, tongue movements, or oral hygiene procedures. Bleeding episodes following these procedures typically occur between postoperative days 3 and 7, when the healing wound remains mechanically vulnerable [12,25]. Consequently, effective hemostasis and local wound protection, as discussed in Section 11, are especially important in open-wound procedures.

By contrast, subepithelial techniques generally allow repositioning of the palatal flap and primary wound closure, providing mechanical coverage and protection of the underlying clot [21,27,43]. Delayed bleeding appears to be uncommon following these procedures [23,24,62,71]. When it occurs, it may result from inadequate intraoperative hemostasis, disruption of the clot beneath the flap, flap instability, or suture loosening. Because the donor site is covered by the repositioned palatal flap, bleeding may initially remain concealed beneath the tissue. Persistent or excessive bleeding may subsequently lead to hematoma formation [12,72].

### 8.3. Swelling

Swelling, nonspecific discomfort, and changes in eating habits or speech are relevant patient-reported outcomes, although they are assessed less consistently than pain and bleeding. These symptoms may be associated with the extent of tissue manipulation, operative duration, concomitant recipient-site surgery, and the degree of mechanical protection provided afforded to the donor-site wound [12].

Patients undergoing open-wound harvesting procedures appear more likely to experience postoperative swelling than those undergoing subepithelial graft harvesting. Smoking has also been associated with an approximately threefold increase in the likelihood of postoperative swelling [25]. In the study by Yen et al., swelling was reported in all participants and was the most frequently reported postoperative complication following SCTG harvesting [73].

### 8.4. Changes in Eating Habits and Masticatory Function

Changes in eating habits may also occur during the early postoperative period [14,24]. These changes may involve modification in food consistency (liquid, soft or solid foods), food temperature, and chewing ability. These outcomes are generally assessed using a 10 cm visual analog scale (VAS) [18].

Greater eating difficulties were reported on postoperative day 3 following DGG harvesting compared with SCTG harvesting [19]. However, this finding should be interpreted cautiously, as temporary dietary modifications are routinely recommended as part of postoperative care to protect the donor site and minimize mechanical trauma.

## 9. Complications Related to Palatal Wound Healing

### 9.1. Clinical Monitoring of Wound Healing

Wound healing monitoring is an important component of postoperative care, as it facilitates the early identification of potential complications. The donor site should be monitored until complete wound healing has been clinically confirmed [12]. Various methods have been used to assess wound healing clinically, including the modified Early Wound Healing Index (EHI), the hydrogen peroxide test to assess epithelialization, VAS-based assessment of tissue color and contour, measurements of wound closure, and visual or computer-assisted analysis of clinical photographs [18].

Complications affecting palatal wound healing should be distinguished from transient symptoms experienced during the early postoperative period. Accordingly, this section focuses not on isolated episodes of pain, bleeding, or discomfort during the first postoperative week, but on persistent or worsening symptoms that may indicate impaired wound healing. Clinically relevant wound-healing complications include delayed epithelialization, wound dehiscence, tissue necrosis, and infection. In the available literature, epithelialization and overall wound healing have been assessed more frequently than dehiscence, necrosis, or infection as distinct clinical outcomes, thereby limiting direct comparisons between studies [12,16,18].

### 9.2. Delayed Wound Healing and Epithelialization

Delayed healing is commonly discussed in the context of palatal donor-site morbidity; however, no standardized definition has been established for palatal donor sites following connective tissue graft harvesting. Studies differ in their choice of outcome measures, assessment time points, and clinical scoring systems [18,22,71].

Nevertheless, available evidence suggests that wound healing may be influenced by wound characteristics, the approach to donor-site closure, and the effectiveness of local wound stabilization. Clinical trials evaluating different methods of wound stabilization and protection—such as suturing technique, collagen sponges, cyanoacrylate tissue adhesives, and platelet-rich fibrin—have generally assessed wound healing in conjunction with postoperative pain and other patient-reported outcomes, rather than specifically investigating delayed or impaired healing as a distinct clinical outcome [62,74,75].

### 9.3. Wound Dehiscence, Flap Sloughing and Necrosis

**Figure 4:**
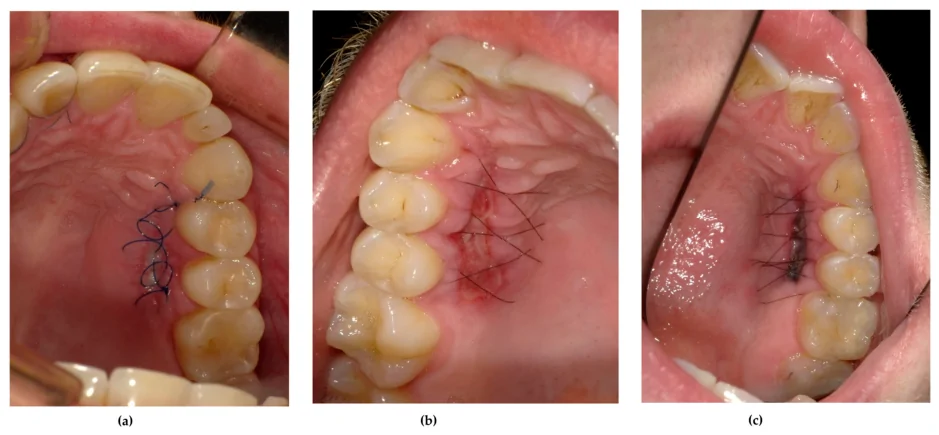
Postoperative complications related to donor-site wound healing: (**a**) flap dehiscence with separation of the incision margins due to premature suture loosening; (**b**) flap sloughing associated with ischemic impairment, resulting in increased wound exposure; and (**c**) flap necrosis with characteristic dark discoloration and tissue breakdown caused by severe ischemia. Clinical photographs taken by A.D.

Wound dehiscence, flap sloughing, and flap necrosis (Figure 4) are clinically relevant complications associated with subepithelial graft-harvesting techniques [7,20,43]. Although palatal wound dehiscence has been infrequently reported, it may occur along sutured incision lines following premature suture loosening or breakage, marginal tissue tearing associated with excessive wound tension, suture placement too close to the wound margins, or traumatic flap handling [6,12]. Dehiscence results in increased wound exposure and may convert a donor site wound intended to heal by primary intention into one that requires partial healing by secondary intention.

Flap sloughing and necrosis are potential ischemic complications associated with excessive thinning of the residual flap or disruption of its vascular supply. Adequate residual flap thickness is important for maintaining tissue perfusion and reducing the risk of necrosis. A minimum residual flap thickness of 1 mm has been recommended, with the flap gradually increasing in thickness from the incision line toward the deeper portion of the palate [22].

Clinically, flap sloughing or necrosis may present with persistent or worsening pain, grayish tissue discoloration, tissue breakdown, malodor, or delayed epithelialization [1]. The reported frequency of partial or complete sloughing or necrosis of the residual palatal flap following SCTG harvesting ranges from 20% to 92% and appears to vary according to the harvesting approach, with a higher prevalence reported following the trap-door technique compared with the single-incision technique [6,7,22].

The severity of wound breakdown may be assessed using the modified EHI, with scores of 4 and 5 indicating severely impaired wound healing. Complete flap necrosis with bone exposure was reported in one of 36 patients in the study by Frikl et al., and occurred in the trap-door group [20]. In the study by Maino et al. which evaluated the single-incision technique, partial or complete necrosis of the residual palatal flap, corresponding to EHI scores of 4 or 5, was observed in 25% of participants at postoperative week 1 [22].

### 9.4. Infection

Infection appears to be uncommon following palatal CTG harvesting [25,66,73]. Worsening pain, suppuration, malodor, fever, progressive swelling or delayed epithelialization should prompt clinical reassessment [12]. In a practice-based study involving 500 patients, infection was reported in only 0.8% of cases [66]. This low reported incidence may partly reflect strict preoperative oral hygiene requirements and the use of antiseptic agents during postoperative care, although the contribution of these factors remains uncertain.

### 9.5. Herpetic Reactivation

Herpetic reactivation triggered by surgical trauma may mimic an early wound-healing complication and should be considered in susceptible patients [12,25].

## 10. Late Donor-Site Complications

Late donor-site complications are defined in this review as adverse symptoms or sequelae that persist or emerge after the expected healing period of the palatal donor site. Despite their potential clinical relevance, persistent sensory disturbances, symptomatic scarring, and palatal surface irregularities remain poorly characterized and underreported in the literature.

### 10.1. Sensory Disturbances

Postoperative sensory disturbances may include loss of sensation, numbness, burning sensations, altered thermal perception, and mechanical hypersensitivity [13,14,76,77]. The dense sensory innervation of the palate, combined with interindividual anatomical variation in the course of the greater palatine nerve, provides a biologically plausible explanation for these disturbances following connective tissue graft harvesting [24,36,78,79,80].

Localized postoperative somatosensory changes are usually transient, with normal sensation typically returning within 4–8 weeks [12,24]. These changes generally do not impair essential oral functions [77].

An electric shock-like sensation affecting the donor site has also been reported, although this symptom was observed in only 1.3% of participants [77]. Similarly, a study involving 500 patients reported a low prevalence of sensory disturbances. Transient paresthesia at the donor site following SCTG harvesting was observed in two patients (0.4%), whereas persistent paresthesia lasting more than 3 months was reported in one patient (0.2%) [66].

The course of sensory recovery may be influenced by the graft-harvesting technique. In a study comparing three approaches—the trap-door (TD), free gingival graft (FGG), and single-incision techniques (SIT)—sensory recovery appeared to be faster in the SIT group. At 2 weeks, normal sensation was restored in 25% of participants in the SIT group, compared with 8% in the TD group and 0% in the FGG group [24]. However, these between-group differences did not reach statistical significance.

In a recent study using objective sensory testing, only thermal sensitivity changed significantly over time, with hypersensitivity observed at 3 months and subsequent hyposensitivity at 6 months. Nonpainful sensory disturbances, particularly numbness, were reported at 18.4% of donor sites [77].

### 10.2. Scarring and Palatal Surface Irregularities

Late scar-related complications have been infrequently reported, as most clinical studies focus on early wound healing and patient-reported morbidity rather than long-term scar morphology or changes in palatal contour [14,22,27].

**Figure 5:**
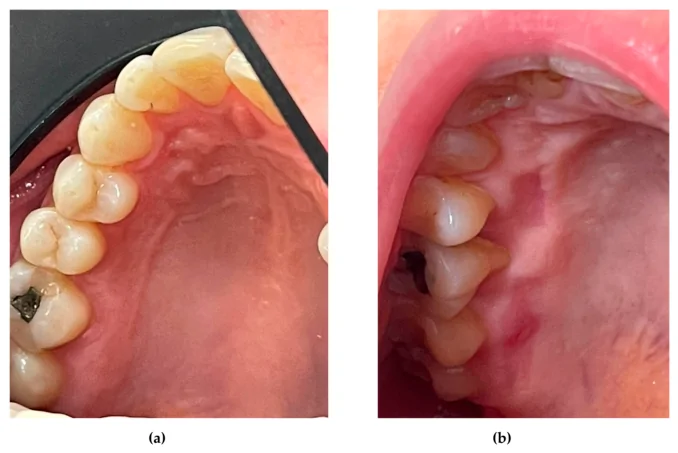
Late donor-site complications following de-epithelialized gingival graft harvesting: (**a**) scar formation; (**b**) irregular palatal contour after harvesting the graft in two separate segments to avoid the palatal root prominence of the maxillary first molar in a periodontal patients. Clinical photographs taken by A.D.

Scarring and palatal surface irregularities (Figure 5) are clinically relevant when they cause symptoms rather than representing merely visible changes. They may manifest as tongue discomfort, food retention, or intolerance of removable dental appliances. Such consequences are clinically plausible, particularly when healing involves extensive tissue loss or occurs by secondary intention. However, their frequency and severity have not been reliably quantified in studies of connective tissue graft harvesting [18].

Overall, late donor-site complications following palatal CTG harvesting remain poorly characterized and infrequently reported in the literature. Current understanding of these complications is informed primarily by biological plausibility, anatomical considerations, and expert clinical interpretation rather than robust long-term quantitative evidence [14,77].

The principal intraoperative, early postoperative, wound-healing, and late donor-site complications associated with palatal connective tissue graft harvesting are summarized in Table 3, together with available data on their frequency and the corresponding levels of evidence.

## 11. Risk-Reduction Strategies for Donor-Site Morbidity and Complications

Given the lack of evidence supporting a single protocol capable of eliminating donor-site morbidity, the term “risk reduction” is a more appropriate than “prevention” in the context of palatal connective tissue graft harvesting. Accordingly, the available recommendations should be regarded as a pragmatic, evidence-informed framework based on palatal anatomy, findings from studies of donor-site morbidity, principles of periodontal plastic surgery, and the limited body of CTG-specific clinical evidence. The interpretation of the available evidence is further complicated by the fact that, although patient-reported outcomes are frequently evaluated, clinically significant complications—including major bleeding, necrosis, infection, and persistent sensory disturbances—remain insufficiently quantified [14,16].

### 11.1. Preoperative Risk Stratification

Risk reduction begins with a comprehensive preoperative risk assessment. The patient’s medical history should be reviewed to identify factors that may increase bleeding risk or impair wound healing, including a history of bleeding episodes, coagulation disorders, antiplatelet or anticoagulant therapy, combined antithrombotic therapy, relevant systemic conditions, smoking, and the use of herbal supplements that may affect hemostasis [50,51,82,83].

However, CTG-specific protocols for the perioperative management of anticoagulant therapy, international normalized ratio (INR) thresholds, and indications for mandatory medical consultation are lacking. Clinical decisions should therefore be individualized based on the patient’s systemic risk, the anticipated extent of the donor-site wound, and the feasibility of achieving adequate local hemostasis.

### 11.2. Anatomy-Guided Planning

Anatomy-guided planning is essential for donor-site safety. Palatal mucosal thickness and the course of the greater palatine artery help define the surgical safety zone and should guide the selection of the harvesting technique, incision placement, and dissection depth [9,10]. Risk-reduction strategies should be tailored to the individual patient based on anatomical findings and should include adherence to evidence-based recommendations and careful palpation to help locate the greater palatine foramen [7].

In patients with thin palatal mucosa or an increased risk of bleeding, as well as in cases requiring a large graft, the surgical plan may need to be modified. Possible modifications include reducing the graft dimensions, selecting an alternative harvesting technique, staging the treatment, or using a soft-tissue substitute. Technique selection should remain individualized, as the available evidence does not support a universal algorithm for choosing between subepithelial and de-epithelialized graft-harvesting approaches [1,6,8,20,21,24].

Harvesting a more superficial DGG may reduce the risk of injury to deeper palatal vessels, whereas deeper dissection during SCTG harvesting may increase the risk of vascular injury and subsequent bleeding [83]. Because SCTG harvesting requires adequate palatal mucosal thickness, limiting the harvesting area to the region anterior to the first molar has been proposed. By contrast, harvesting a free mucosal graft for subsequent de-epithelialization involves shallower incisions and may be extended posterior to the first molar, with a lower risk of GBA injury [8].

### 11.3. Strategies for Postoperative Pain Control

The graft-harvesting technique may also influence the risk of severe postoperative pain. Healing by primary intention at an SCTG donor site may reduce this risk, provided that adequate residual flap thickness is maintained and flap sloughing or necrosis is avoided. However, Zucchelli et al. [6] reported no statistically significant difference in patient-reported postoperative pain between DGG harvesting with appropriate protection of the open palatal wound and SCTG harvesting using the trap-door technique.

Local therapeutic interventions, including platelet-rich fibrin, hyaluronic acid, and enamel matrix derivatives, may help reduce postoperative pain and discomfort while promoting wound healing and epithelialization following open-wound graft harvesting [84,85,86].

A network meta-analysis ranked leucocyte- and platelet-rich fibrin (L-PRF) highest among the evaluated intervention for postoperative pain reduction. On postoperative day 1, the interventions were ranked as follows: L-PRF, ozone therapy, laser therapy, suturing, and hemostatic sponges. On day 3, L-PRF remained the highest-ranked intervention, followed by suturing, laser therapy, ozone therapy, and hemostatic sponges. On day 7, L-PRF continued to rank highest for postoperative pain control [87]. Table 4 summarizes systematic reviews evaluating therapeutic approaches that may reduce postoperative pain and support donor-site wound healing following palatal graft harvesting.

### 11.4. Intraoperative Tissue Management

Intraoperative risk reduction depends on minimizing avoidable tissue trauma, achieving effective and sustained hemostasis, and ensuring wound stability. Graft dimensions should be limited to the minimum necessary to achieve the treatment objectives at the recipient site. Careful control of dissection depth, preservation of adequate residual flap thickness when a covering flap is retained, atraumatic tissue handling, and appropriate wound stabilization are particularly important during palatal graft harvesting [22].

### 11.5. Hemostasis and Local Wound Stabilization

Adequate hemostasis and wound stability should be achieved before completion of the surgical procedure. Initial management typically involves applying direct pressure with moistened gauze for approximately 5 min, followed by suturing if necessary [52,54]. The risk of intraoperative bleeding may also be reduced by placing a greater palatine compression suture before graft harvesting [30]. Bleeding that persists despite these measures should be regarded as an intraoperative complication requiring further hemostatic intervention [18].

Adjunctive interventions have been proposed to facilitate hemostasis at the donor site, particularly in wounds healing by secondary intention. These interventions include collagen or gelatin sponges, oxidized regenerated cellulose, plant-derived extracts, and cyanoacrylate tissue adhesives [56,62,63,74,101,102,103,104]. As with postoperative pain, a network meta-analysis ranked L-PRF highest among the evaluated interventions for reducing postoperative bleeding following open-wound graft harvesting [87].

The risk of delayed bleeding, particularly during the early healing phase of an open palatal wound, may also be reduced by protecting the donor site with periodontal dressings, flowable composite resin, or a palatal stent. However, palatal stent use has been associated with increased analgesic consumption in some studies [14,16].

Although these adjunctive measures may improve clot stability and wound protection, their effects on patient comfort may vary. Furthermore, the available evidence is heterogeneous and frequently comes from studies of FGG or DGG donor sites, limiting its direct applicability to SCTG donor sites [14,16,87]. Local hemostatic and therapeutic interventions should therefore be selected based on wound morphology, bleeding characteristics, and patient-related risk factors [62,63,70,74,100,102,104].

### 11.6. Postoperative Instructions and Patient Adherence

Postoperative instructions are an important component of risk reduction, although evidence regarding their optimal content and the recommended duration of specific postoperative restrictions following SCTG harvesting remains limited. Patients should receive both written and verbal instructions concerning wound protection, dietary modifications, modification to oral hygiene practices, the use of a palatal stent when indicated, the management of postoperative pain and bleeding, and warning signs that warrant clinical reassessment.

Overall, the risk of donor-site morbidity and complications may be reduced through a combination of thorough preoperative risk assessment, anatomy-guided planning of the harvesting procedure, appropriate technique selection, atraumatic tissue handling, effective hemostasis, wound stabilization and protection, and patient adherence to postoperative instructions.

## 12. Management of Postoperative Complications

The management strategies described in this section represent a pragmatic clinical framework rather than a validated treatment algorithm. This distinction reflects the fragmented nature of the available evidence, as not all data are derived directly from studies of donor sites following SCTG or DGG harvesting. Some recommendations are extrapolated from studies of FGG donor sites, anatomical studies of the palate, and general principles of periodontal and oral surgery [6,9,12,17,27,50,64,87]. Moreover, severe complications—such as arterial injury, extensive necrosis, pseudoaneurysm, and persistent sensory disturbances—are infrequently reported and remain underrepresented in randomized clinical trials.

A practical management sequence should include recognition of the complication, assessment of its severity, and identification of potential local causes or contributing factors. The clinician should then determine whether continued observation is appropriate or escalation of care is required, followed by appropriate follow-up planning. This structured approach is particularly important because the same clinical finding—such as pain or incomplete epithelialization—may reflect normal healing, mechanical irritation, clot instability, or infection, depending on its timing, severity, and associated clinical features.

### 12.1. Management of Bleeding

Delayed bleeding should be managed according to its source, severity, and timing. Minor oozing should be distinguished from more significant bleeding, and clinical assessment should consider potential causes, such as focal vascular injury, clot disruption, or stent-related trauma, as well as signs of an expanding hematoma. Minor bleeding can generally be controlled with firm direct pressure applied using moistened gauze for 10–20 min.

Persistent, recurrent, or pulsatile bleeding requires prompt clinical intervention. Initial management should include direct compression and identification of the bleeding source. Further measures may include administration of a local anesthetic containing a vasoconstrictor when not contraindicated, placement of compression sutures, carefully targeted electrocautery, or application of local hemostatic materials [19,50,57,70].

Hemostatic methods should not be regarded as interchangeable, as they serve different functions. Sutures stabilize tissues and may provide compression, collagen or gelatin sponges support clot formation, cyanoacrylate tissue adhesives provide a superficial protective barrier, and palatal stents may provide mechanical protection and compression. In patients receiving antiplatelet or anticoagulant therapy, local hemostasis remains central; however, any proposed modification of antithrombotic therapy should be individualized and coordinated with the prescribing physician when necessary.

### 12.2. Management of Postoperative Pain

Pain should be interpreted in relation to the expected postoperative course. Pain that progressively decreases and is consistent with the clinical appearance of the wound can generally be managed symptomatically. Conversely, pain that increases after an initial improvement, becomes throbbing, appears disproportionate to the clinical findings, or is associated with malodor, progressive swelling, suppuration, bone exposure, or necrosis should prompt clinical reassessment, as discussed below.

The available literature does not support a universal CTG-specific protocol for postoperative analgesia [14]. Analgesic regimens following surgical procedures involving palatal graft harvesting most commonly include non-opioid medications, such as paracetamol (acetaminophen) and nonsteroidal anti-inflammatory drugs (NSAIDs), particularly ibuprofen [6,19,23]. Topical flurbiprofen has also been proposed as an adjunctive treatment [85]. The use of NSAIDs should be individualized based on the patient’s bleeding risk and gastrointestinal, renal, and cardiovascular risk factors. Opioid analgesics are rarely required and should be reserved for selected cases of severe pain that cannot be adequately controlled with non-opioid analgesics [105].

### 12.3. Delayed Healing and Suspected Infection

Delayed healing should be suspected when the degree of epithelialization, wound contraction, or flap integrity is clearly inconsistent with the expected healing course for the harvesting technique used. Initial management should address potential contributing factors, including plaque accumulation, smoking, mechanical irritation, clot instability, excessive pressure from a palatal stent, and premature loss of the wound dressing [7].

Slow but otherwise uncomplicated epithelialization does not warrant routine antibiotic therapy. By contrast, suppuration, increasing erythema, progressive swelling, malodor, or fever should raise suspicion of infection. Management may include local debridement, saline irrigation, the use of a topical antiseptic, and—only when clinically indicated—systemic antibiotic therapy [12,106].

### 12.4. Wound Dehiscence and Necrosis

Wound dehiscence and necrosis should be assessed according to their extent and progression. Limited superficial defects may be managed conservatively by eliminating local trauma, maintaining adequate plaque control with adjunctive chlorhexidine rinses, and ensuring close clinical follow-up.

Persistent infection or extensive necrosis with bone exposure warrants prompt surgical reassessment and may necessitate debridement, additional suturing when appropriate, or adjustment of the palatal stent [20,107]. However, evidence supporting specific management protocols remains limited.

### 12.5. Persistent Sensory Disturbances

Persistent sensory disturbances should be documented by recording their location, extent, and duration. Clinical monitoring for spontaneous resolution is appropriate in most cases, whereas persistent or progressive symptoms may warrant specialist referral for further evaluation [77].

## 13. Discussion

The principal conclusion of this narrative review is that palatal donor-site morbidity is influenced not only by the graft-harvesting technique, but by the interplay of multiple factors. These include patient susceptibility, palatal anatomy, graft requirements, wound configuration, surgical execution, and postoperative care. A mechanism-based interpretation helps distinguish expected postoperative symptoms from true complications and the inherent biological burden of surgery from modifiable technical factors.

Complications following palatal graft harvesting should not automatically be attributed to technical errors. Some adverse events may occur despite appropriate surgical planning, consideration of anatomical factors, and careful performance of the procedure, particularly in patients with anatomical variability, thin palatal mucosa, poor plaque control, or systemic conditions, as well as in those who smoke or take medications that affect bleeding risk or wound healing [9,25,51,82].

Nevertheless, technical errors are clinically important because they represent potentially modifiable contributors to morbidity. Excessive dissection depth, inadequate residual tissue or flap thickness, ineffective hemostasis, and inadequate wound protection may increase the risk or severity of postoperative complications [6,7,21,54,67,106]. Conversely, mild pain or minor oozing may occur despite technically appropriate surgery. Pain and bleeding are the most frequently assessed outcomes, whereas wound dehiscence, necrosis, infection, sensory disturbances, and symptomatic scar-related changes are evaluated less consistently [13,14,18,25].

The available evidence therefore supports an anatomy-guided and wound-specific, rather than exclusively technique-driven, approach to donor-site safety. Palatal mucosal thickness, site-specific tissue composition, required graft dimensions, and the course of the greater palatine neurovascular bundle help define the extent of the usable surgical field and its safety margins [8,9,10,11,21]. However, interindividual and intraindividual anatomical variability means that neither a fixed “safe zone” nor a universally appropriate graft thickness can be assumed. The smallest graft capable of achieving the treatment objective at the recipient site should be harvested, with incision placement, dissection depth and harvesting technique adapted to the available tissue volume and vascular anatomy [1,21,108]. A thicker palate does not necessarily constitute a more favorable donor site, as greater mucosal thickness may reflect a higher proportion of adipose or glandular tissue rather than dense connective tissue [8].

Palatal graft-harvesting techniques should also be considered in relation to the wound configurations they create. Subepithelial approaches preserve epithelial coverage and are intended to achieve primary wound closure, whereas DGG harvesting creates an open wound that heals by secondary intention [6]. Differences in postoperative pain, bleeding, and epithelialization may therefore reflect differences in wound biology. Closed-wound approaches may reduce wound exposure but require adequate control of dissection depth and graft thickness, as well as preservation of a viable residual flap. Open techniques facilitate precise control over graft dimensions but require meticulous hemostasis, appropriate wound protection, and close monitoring of the wound healing [6,13,22,62].

As with all surgical procedures, complete elimination of donor-site morbidity following graft harvesting is not possible. However, several measures may reduce its risk and severity. These include identification of systemic and anatomical risk factors, determination of the minimum effective graft dimensions, selection of the harvesting technique according to tissue availability and the anticipated wound configuration, preservation of adequately vascularized residual tissue, achievement of effective hemostasis before patient discharge, and provision of wound-specific postoperative instructions, including information on warning signs, that warrant urgent clinical reassessment.

Adjunctive interventions—such as compression sutures, hemostatic sponges, cyanoacrylate tissue adhesives, platelet-rich fibrin, and hyaluronic acid—may support clot stability, wound healing, or patient comfort, depending on the intervention, but cannot compensate for inadequate surgical planning or traumatic tissue handling [18,62,70,74,87,100,102].

The available evidence remains heterogeneous with respect to graft-harvesting techniques, graft dimensions, wound-closure methods, pharmacological regimens, and follow-up duration [14,16,18]. Severe complications and long-term neurosensory outcomes are infrequently assessed as primary endpoints. Furthermore, many risk-reduction principles are based on anatomical evidence and established surgical principles rather than on clinical trials specifically designed to assess the contribution of individual technical factors to donor-site complications following different harvesting techniques.

This review is limited by its narrative design, restriction to full-text English-language publications, and the absence of a formal risk-of-bias assessment and a meta-analysis. Evidence from FGG donor sites was included when DGG-specific data were unavailable; however, such evidence may not be directly applicable to DGG harvesting or to SCTG donor sites that heal by primary intention. These limitations preclude reliable quantitative comparison and ranking of graft-harvesting techniques or adjunctive interventions.

## 14. Conclusions

Nevertheless, a consistent clinical conclusion is that donor-site morbidity following palatal connective tissue graft harvesting may be reduced but cannot be completely eliminated. Donor-site outcomes reflect the interplay of graft requirements, individual palatal anatomy, and potentially modifiable factors related to surgical planning, technical execution, and operator experience.

Pain and bleeding are among the most frequently assessed early postoperative outcomes, whereas wound infection, flap necrosis, persistent sensory disturbances, and symptomatic scar-related changes are clinically important but remain insufficiently quantified.

No single harvesting technique or adjunctive material has been demonstrated to be universally superior. Technique selection should balance recipient-site requirements with the anticipated donor-site wound configuration and healing pattern. The selected approach should aim to provide sufficient tissue for the recipient site while minimizing donor-site morbidity and promoting patient acceptability.

In clinical practice, the risk of postoperative morbidity and complications may be reduced through a structured perioperative approach comprising medical and anatomical assessment, determination of the minimum graft dimensions required to achieve the treatment objectives, atraumatic tissue handling, secure hemostasis, technique-specific wound protection, clear postoperative instructions, and timely clinical reassessment when warning signs arise.

Because the available evidence is heterogeneous and focuses largely on short-term patient-reported outcomes, these principles should be applied as evidence-informed guidance in everyday clinical practice rather than as a validated universal treatment algorithm.

## 15. Future Direction

Future research on palatal connective tissue graft harvesting should prioritize standardization and clinical comparability rather than merely increasing the number of interventional trials. In our view, establishing a core outcome set should be a priority to enable consistent differentiation between expected postoperative symptoms and clinically relevant complications and to establish reproducible definitions, assessment methods, and follow-up intervals for bleeding severity, delayed epithelialization, necrosis, infection, functional impairment, and neurosensory disturbances.

Prospective studies should investigate potentially modifiable surgical variables, including graft dimensions, residual flap thickness, wound configuration, and clot stability, in relation to individual palatal anatomy, systemic conditions, and concomitant medication use. We also suggest that adjunctive interventions should be classified according to their primary mechanisms of action and evaluated separately for primarily closed and open palatal donor-site wounds.

We propose that future research should focus on developing and validating CTG-specific risk-stratification tools and practical algorithms for harvesting-technique selection, integrating anatomical characteristics, patient-related risk factors, and standardized postoperative outcomes. Such approaches could support individualized surgical planning and help identify patients requiring closer postoperative monitoring.

Finally, future research should evaluate standardized preoperative checklists, morbidity-focused informed-consent procedures, and patient-education protocols that incorporate clearly defined criteria for early clinical reassessment. In our view, prospective evaluation of these integrated care pathways, rather than isolated interventions, represents a promising approach to developing evidence-based strategies for reducing donor-site morbidity, lowering the risk of significant complications, and improving their management.

## Figures and Tables

**Table 1:** Characteristics of the key studies included in the narrative literature review.

Author and Year	Study Design	Objective	Sample Size/ Studies Included	Graft Type/Harvesting Technique	Outcome Assessment	Main Donor-Site Findings
Palatal anatomy						
Tomaszewska et al., 2014 [17]	Systematic review and meta-analysis.	To assess GPF location relative to anatomical landmarks, particularly maxillary molars.	27 studies in the systematic review; 23 studies in the meta-analyses (6927 GPFs).	50 adult skulls and 1200 head CT scans.	GPF location.	GPF most frequently opposite the third molar (63.6%); maxillary molars were identified as the most reliable landmarks.
Tavelli et al., 2019 [9]	Systematic review.	To define a safety zone for palatal graft harvesting.	26 studies; (5768 hemipalates).	Cadaveric, CT, and CBCT studies.	GPF location and GPA course relative to maxillary teeth.	An anatomically based safety zone for palatal graft harvesting was proposed.
Complications and postoperative morbidity						
Malpartida-Carrillo et al., 2014 [18]	Systematic review.	To summarize outcome measures for palatal wound healing and patient-reported morbidity.	31 studies.	Free gingival grafts (FGGs) and connective tissue grafts (CTGs).	Palatal wound healing and patient-reported morbidity (pain, analgesic use, discomfort, quality of life, burning sensation, and changes in eating habits).	Common healing assessments: hydrogen peroxide test, tissue color match, visual inspection, and bleeding. Most frequently assessed patient-reported outcomes: pain, analgesic use, discomfort, burning sensation, and changes in eating habits.
Chong et al., 2025 [14]	Systematic review.	To assess postoperative pain and donor-site morbidity following palatal graft harvesting and wound-dressing interventions.	69 studies.	Free gingival graft (FGG) and de-epithelialized gingival graft (DGG) techniques.	Pain, quality of life, bleeding, burning sensation, sensory changes, and eating difficulties.	Pain peaked during the first 3 days and decreased thereafter. Wound dressings and physical barriers may reduce pain, with effects varying by intervention.
Figueras-Alvarez et al., 2026 [13]	Systematic review.	To assess complications following palatal soft-tissue graft harvesting.	16 studies.	Autologous soft-tissue graft.	Wound healing, pain, discomfort, bleeding, and sensory disturbances.	Pain was the most common complication, generally mild and transient. Adjunctive interventions may reduce discomfort and support healing. Bleeding and sensory disturbances were less frequent and generally transient.
Mashaly et al., 2002 [19]	Randomized, controlled trial.	To compare patient morbidity and root-coverage outcomes following CAF + DGG versus CAF + SCTG.	28 Egyptian patients: CAF + DGG (n = 14); CAF + SCTG (n = 14).	Single-incision SCTG; extraorally de-epithelialized DGG.	Pain, stress, bleeding, chewing difficulty, and satisfaction; assessed at days 3, 7, and 14.	CAF + DGG was associated with significantly greater stress and chewing difficulty at day 14 compared with CAF + SCTG.
Zucchelli et al., 2010 [6]	Two-center, double-blind, parallel-group RCT.	To compare patient morbidity and root-coverage outcomes following CAF + DGG versus CAF + SCTG.	50 Italian patients: CAF + DGG (n = 25); CAF + SCTG (n = 25).	Trap-door SCTG; DGG.	Palatal and graft thickness; analgesic use, discomfort, bleeding, stress, and chewing difficulty; assessed at 1 week.	No significant differences in analgesic use, discomfort, or bleeding. SCTG was associated with less stress and chewing difficulty. Analgesic use increased with graft height and flap dehiscence or necrosis; pain was inversely correlated with residual palatal tissue thickness.
Fickl et al., 2014 [20]	Randomized, single-blinded controlled trial.	To compare wound healing and pain following SIT, mSIT, and TD harvesting techniques.	36 German patients: SIT (n = 12); mSIT (n = 12); TD (n = 12).	Single-incision (SIT), modified single-incision (mSIT), and trap-door (TD) SCTG harvesting.	Early Wound Healing Index (EHI), VAS pain scores, and analgesic use (dose and duration); assessed at 1 week.	SIT and mSIT showed better early wound healing, less secondary healing, and significantly lower cumulative analgesic doses and shorter duration of analgesic use than TD.
Zucchelli et al., 2014 [21]	Randomized, double-blind, parallel-group RCT.	To assess the effect of graft thickness and height on patient morbidity.	60 Italian patients: reduced-size DGG (n = 30; thickness < 2 mm, height 4 mm) vs. conventional DGG (n = 30; thickness ≥ 2 mm, height corresponding to bone dehiscence).	Extraorally de-epithelialized gingival graft (DGG).	Analgesic consumption, discomfort, bleeding, and chewing difficulty; assessed at 1 week.	Reduced graft thickness and height decreased patient morbidity without compromising root-coverage outcomes.
Maino et al., 2018 [22]	Randomized, single-blind clinical trial.	To assess the effects of suturing technique and palatal tissue thickness on wound healing and pain after SCTG harvesting.	36 Spanish patients: continuous interlocking sutures (n = 18) vs. interrupted criss-cross sutures (n = 18).	Single-incision SCTG harvesting.	Preoperative mucosal and residual flap thickness; EHI at day 7; VAS pain scores at days 1–28.	Residual flap thickness was significantly associated with EHI and inversely correlated with days of discomfort. Suturing technique showed no significant association with wound healing.
Bakhishov et al., 2021 [23]	Single-blind, parallel-group RCT.	To compare clinical outcomes and morbidity following DGG + TUN versus SCTG + TUN.	27 Turkish patients: DGG + TUN (n = 13); SCTG + TUN (n = 14).	Intraorally de-epithelialized DGG; single-incision SCTG.	Donor-site pain, discomfort, sensitivity, and bleeding; assessed at days 1–28.	No significant between-group differences in postoperative pain, discomfort, sensitivity, or systemic analgesic use.
Del Pizzo et al., 2002 [24]	Preliminary comparative clinical study.	To compare early palatal wound healing following SI, FGG, and TD harvesting techniques.	36 Italian patients: SI (n = 12), FGG (n = 12), TD (n = 12).	Single-incision SCTG, trap-door SCTG, and FGG harvesting.	Bleeding, sensitivity, epithelialization, discomfort, and changes in eating habits; assessed at weeks 1–8.	Complete epithelialization occurred significantly earlier after SCTG harvesting than after FGG harvesting. Discomfort was significantly lower with SI and TD than with FGG.
Griffin et al., 2006 [25]	Prospective observational clinical study.	To compare postoperative complications following FSTG and SCTG harvesting and identify potential risk factors.	228 American patients (242 procedures): FSTG (n = 70); SCTG (n = 172).	Full-thickness SCTG (Bruno technique); FSTG.	Pain (0–10 numerical scale), swelling, and bleeding; assessed at 1 week.	Longer surgical duration was associated with pain and swelling. Smoking was associated with a threefold higher likelihood of swelling. FSTG was associated with approximately threefold higher likelihood of moderate-to-severe pain and bleeding compared with SCTG.
Wessel and Tatakis, 2008 [26]	Prospective observational parallel-group study.	To compare patient-reported morbidity following CTG and FGG harvesting.	23 American patients: CTG (n = 12); FGG (n = 11).	CTG (Harris or Hürzeler and Weng technique); FGG with palatal stent.	VAS pain scores, analgesic consumption, and duration of analgesic use; assessed at day 3 and week 3.	FGG harvesting was associated with a higher incidence of early postoperative donor-site pain than CTG harvesting.
Lessa et al., 2025 [27]	Prospective cohort study.	To compare donor-site morbidity following DGG and SCTG harvesting.	16 Brazilian patients: DGG (n = 8); SCTG (n = 8).	DGG; single-incision SCTG.	VAS pain scores, medication use, and wound healing; assessed at weeks 1–3.	DGG was associated with greater postoperative pain and higher analgesic and anti-inflammatory medication requirements than SCTG.
Post-surgical management of donor site						
de Almeida et al., 2024 [15]	Umbrella review.	To evaluate interventions for palatal wound healing and postoperative morbidity.	10 systematic reviews (25 RCTs).	DGG and SCTG.	Wound healing, pain, bleeding, and analgesic consumption.	LLLT, PRF, CTAs, and ozone therapy showed potential benefits for wound healing. LLLT, PRF, and CTAs reduced pain and analgesic use; PRF and CTAs reduced bleeding. Review quality: 7 low/critically low, 2 moderate, and 1 high
Leite et al., 2025 [16]	Systematic review.	To evaluate pain-management interventions and their effects on oral health-related quality of life (OHRQoL) after palatal graft harvesting.	54 studies (43 different interventions).	FGG; DGG.	Postoperative pain and the OHRQOL	Postoperative pain generally decreased over time. No superior intervention could be identified due to heterogeneity and the use of combined interventions with different mechanisms of action.
Ziyaettin et al., 2026 [28]	Double-blind RCT.	To evaluate the effects of pre-harvesting GPCS on bleeding, morbidity, wound healing, and quality of life.	32 Turkish patients: GPCS (n = 16); control (n = 16).	Single-incision SCTG harvesting, with or without preoperative GPCS.	Bleeding, surgical duration, pain, analgesic use, Landry healing index, and OHIP-14; assessed from day 3 to month 3.	GPCS reduced intraoperative bleeding and surgical duration, with favorable effects on postoperative morbidity and wound healing.

**Table 2:** Selected milestones in the evolution of palatal connective tissue graft harvesting techniques.

Author(s) and Year	Harvesting Technique	Key Surgical Characteristics	Donor-Site Wound Configuration	Potential Implications for Donor-Site Morbidity
Edel, 1974 [37]	Trap-door technique	Horizontal and vertical incisions; partial- or full-thickness flap elevation; CTG harvested beneath or from the reflected flap.	Repositioned and sutured palatal flap; primary wound closure.	Good surgical visibility; vertical incisions may compromise flap vascularity and increase the risk of sloughing.
Langer and Langer, 1982 [38]	Subepithelial CTG harvesting with parallel incisions	Parallel horizontal incisions combined with vertical incisions; CTG may include an epithelial collar.	Sutured donor site; an exposed area may remain and heal by secondary intention.	Incomplete wound closure and interruption of flap vascularity may increase postoperative morbidity.
Raetzke, 1985 [41]	Envelope technique	Two converging horizontal incisions used to harvest a wedge-shaped graft without vertical incisions.	Relatively limited surface wound; sutured margins, although an epithelial donor area may remain exposed.	Avoidance of vertical incisions may preserve vascularity; graft dimensions are limited, especially in thin palatal mucosa.
Harris, 1992 [42]	Parallel-blade and Unigraft knife techniques	Specialized instruments used to obtain CTGs with controlled thickness.	Sutured wound; incomplete closure may occur when an epithelial collar is included.	Improved graft thickness control, but limited adaptability to thin palatal mucosa and possible residual wound exposure.
Bruno, 1994 [39]	Double-incision technique	Two horizontal incisions without vertical releasing incisions; graft separated using a periosteal elevator.	Sutured wound; complete closure may be difficult.	Avoidance of vertical incisions may preserve blood supply; limited access and residual exposure remain potential concerns.
Hürzeler and Weng, 1999 [40]	Single-incision technique	One horizontal incision with undermining preparation of a split-thickness flap and subepithelial graft dissection.	Repositioned and sutured flap; primary-intention healing.	Reduced surface incisions, but limited visibility and possible bleeding from the initial deep incision.
Lorenzana and Allen, 2000 [43]	Single-incision technique	CTG and periosteum elevated through a single horizontal incision after split-thickness flap preparation.	Sutured flap; primary-intention healing.	Limited wound exposure, but restricted visibility and access may complicate harvesting.
Zucchelli et al., 2003/2010 [6,44]	Extraoral de-epithelialization of a free gingival graft (DGG)	Free gingival graft harvested from the palate and de-epithelialized extraorally.	Open palatal wound healing by secondary intention.	Direct wound exposure and secondary-intention healing may contribute to postoperative discomfort.
McLeod et al., 2009 [45]	In situ de-epithelialization technique	Palatal surface de-epithelialized before harvesting CTG using an FGG-like approach.	Exposed palatal wound healing by secondary intention.	Enables harvesting of larger grafts; wound exposure and bleeding, particularly near the palatal midline, are potential concerns.
Bhatavadekar and Gharpure, 2018 [46]	Controlled palatal harvest technique	L-shaped incision and flap elevation; CTG dissected from the reflected flap with control of residual flap thickness.	Repositioned flap secured with sutures or collagen tape.	Better control of residual flap thickness may reduce flap sloughing; flap perforation and bleeding remain potential risks.

**Table 3:** Summary of donor-site morbidity and complications following palatal connective tissue graft harvesting.

Complication/Donor-Site Event	Clinical Presentation	Typical Timing	Reported Frequency/ Incidence	Evidence Level and Comments	Key References
Intraoperative complications					
Arterial bleeding/suspected vascular injury	Pulsatile bleeding; persistent bleeding despite compression; difficulty achieving hemostasis.	Intraoperative	Not reliably quantified	A case series describes the management of palatal bleeding during FGG harvesting. An RCT assessed intraoperative bleeding but did not establish the incidence of confirmed GPA injury.	[28,57]
Early postoperative morbidity and complications					
Postoperative pain	Expected postoperative pain or pain disproportionate to the stage of healing; may affect quality of life.	Typically peaks on postoperative days 1–3; gradually subsides, usually resolving within approximately 2 weeks.	Common; exact incidence is not consistently reported, as studies more frequently assess pain intensity and analgesic consumption.	Frequently evaluated in clinical studies; reported pain severity may vary with graft dimensions and thickness, wound exposure and protection, postoperative analgesic protocols, and pain assessment scales.	[13,14,18,19]
Delayed bleeding	Bleeding occurring or recurring after surgery, ranging from minor oozing to prolonged bleeding requiring urgent clinical intervention.	Early postoperative period; may occur between postoperative days 3 and 7.	Infrequently reported following SCTG harvesting; 3.0% in a 500-case CTG series; another study reported 17 bleeding episodes in 15 patients.	Less frequently reported than postoperative pain but clinically important; reported frequency may vary according to the harvesting technique and donor-site wound protection.	[13,25,66]
Delayed severe palatal hemorrhage/GPA pseudoaneurysm	Recurrent or severe palatal bleeding; GPA pseudoaneurysm in a documented case.	Several days postoperatively	Not reliably quantified; isolated case reports.	Case-report-level evidence of delayed severe bleeding following SCTG harvesting; GPA pseudoaneurysm confirmed in one published case.	[72,81]
Swelling/edema	Local palatal swelling	Early postoperative period	Infrequently reported as a distinct donor-site outcome; 5.4% in a 500-case CTG series.	Generally mild; donor-site-specific swelling is inconsistently reported in clinical studies.	[13,23,25,66]
Functional discomfort	Difficulties with eating, speaking, swallowing, or performing oral hygiene.	First few postoperative days	Not consistently quantified; generally assessed as temporary functional limitations.	Greater eating difficulties were reported on postoperative day 3 following DGG harvesting than following SCTG harvesting. Functional limitations may also reflect postoperative dietary recommendations or adaptation to protective stents rather than complications.	[14,18,19,24]
Complications related to palatal wound healing					
Delayed epithelialization/delayed wound healing	Delayed epithelial coverage or wound closure relative to the expected healing course.	Usually assessed during the first 2–4 postoperative weeks	Not reliably quantified; definitions and assessment time points vary across studies	Clinical studies assess epithelialization and wound healing, but delayed healing is not consistently reported as a distinct complication. Impaired healing should be distinguished from infection.	[7,23,62]
Partial or complete flap sloughing or necrosis	Flap discoloration, tissue sloughing, localized partial or complete necrosis, possible bone exposure, and delayed wound healing.	Early postoperative period, usually within the first 1–3 weeks.	Reported rates range from 20% to 92% in selected studies of SCTG harvesting; the overall incidence remains uncertain.	Reported rates vary considerably across studies. Flap sloughing or necrosis may be associated with impaired tissue perfusion; severe cases may involve bone exposure.	[6,7,22]
Infection	Suppuration, progressive erythema or swelling, malodor, fever, and wound breakdown.	Early postoperative period	0.8% in one CTG cohort; no clinically significant infections reported in a recent systematic review.	Infrequently reported; may impair wound healing and compromise primary wound closure.	[13,25,66]
Late donor-site complications					
Sensory disturbances/persistent paresthesia	Loss of sensation, numbness, burning sensations, mechanical hypersensitivity, or electric shock-like sensations.	Onset in the early postoperative period; symptoms may persist for weeks or months.	Inconsistently reported; nonpainful sensory disturbances in 18.4% of donor sites in one study; transient paresthesia in 0.4% and persistent paresthesia in 0.2% of patients in a 500-case series.	Usually transient; persistent sensory disturbances are less well characterized, and standardized CTG-specific follow-up intervals are lacking.	[13,24,66,77]
Scarring/palatal surface irregularities	Fibrotic scarring, tissue color changes, and irregular palatal contour	Late postoperative period	Not consistently quantified	Often asymptomatic; clinically relevant when associated with discomfort or functional impairment. Long-term evidence remains limited.	[7,18,21]

**Table 4:** Systematic reviews of therapeutic approaches to enhance wound healing following palatal graft harvesting.

Therapeutic Approach	Key References	Main Findings
Low -level laser therapy (LLLT)/photobiomodulation (PBM)	[88,89,90,91]	LLT/PBM was associated with statistically significant improvements in early wound healing and a short time to complete wound epithelialization compared with spontaneous healing. However, findings regarding postoperative pain reduction and analgesic consumption were heterogeneous across studies.
Platelet-rich fibrin (PRF)	[92,93,94,95,96]	PRF was associated with faster wound healing, reduced postoperative pain, lower analgesic consumption, and a marked reduction in immediate postoperative bleeding.
Ozone therapy (OT)	[97]	OT was associated with improved wound healing.
Cyanoacrylate tissue adhesives (CTAs)	[98,99]	CTAs were associated with faster wound healing, improved control of immediate bleeding, reduced postoperative pain, and lower analgesic consumption.
Hyaluronic acid (HA)	[100]	HA was associated with improved wound epithelialization and color matching, as well as reduced patient-reported postoperative pain.

## Data Availability

No new data were created in this study. Data sharing is not applicable.

## Abbreviations

The following abbreviations are used in this manuscript:

CTG	Connective tissue graft
SCTG	Subepithelial connective tissue graft
DGG	De-epithelialized gingival graft
FGG	Free gingival graft
GPF	Greater palatine foramen
GPB	Greater palatine bundle
GPA	Greater palatine artery
CT	Computed tomography
CBCT	Cone bean computed tomography
RCT	Randomized control clinical trial
GPCS	Greater palatine compression suture
CAF	Coronally advanced flap
TUN	Tunnel technique
TD	Trap-door technique
SIT	Single-incision technique
VAS	Visual analog scale
EHI	Early wound healing index
PROs	Patient-reported outcomes
LLLT	Low-level laser therapy
PBM	Photobiomodulation
PRF	Platelet-rich fibrin
L-PRF	Leucocyte- and platelet-rich fibrin
OT	Ozone therapy
CTAs	Cyanoacrylate tissue adhesives
HA	Hyaluronic acid
NSAIDs	Nonsteroidal anti-inflammatory drugs
